# d-Tubocurarine and Berbamine: Alkaloids That Are Permeant Blockers of the Hair Cell's Mechano-Electrical Transducer Channel and Protect from Aminoglycoside Toxicity

**DOI:** 10.3389/fncel.2017.00262

**Published:** 2017-09-05

**Authors:** Nerissa K. Kirkwood, Molly O'Reilly, Marco Derudas, Emma J. Kenyon, Rosemary Huckvale, Sietse M. van Netten, Simon E. Ward, Guy P. Richardson, Corné J. Kros

**Affiliations:** ^1^Sussex Neuroscience, School of Life Sciences, University of Sussex Brighton, United Kingdom; ^2^Sussex Drug Discovery Centre, School of Life Sciences, University of Sussex Brighton, United Kingdom; ^3^Institute of Artificial Intelligence and Cognitive Engineering, University of Groningen Groningen, Netherlands

**Keywords:** hair cell, mechanotransduction, hearing loss, ototoxicity, aminoglycosides, d-tubocurarine, berbamine

## Abstract

Aminoglycoside antibiotics are widely used for the treatment of life-threatening bacterial infections, but cause permanent hearing loss in a substantial proportion of treated patients. The sensory hair cells of the inner ear are damaged following entry of these antibiotics via the mechano-electrical transducer (MET) channels located at the tips of the hair cell's stereocilia. d-Tubocurarine (dTC) is a MET channel blocker that reduces the loading of gentamicin-Texas Red (GTTR) into rat cochlear hair cells and protects them from gentamicin treatment. Berbamine is a structurally related alkaloid that reduces GTTR labeling of zebrafish lateral-line hair cells and protects them from aminoglycoside-induced cell death. Both compounds are thought to reduce aminoglycoside entry into hair cells through the MET channels. Here we show that dTC (≥6.25 μM) or berbamine (≥1.55 μM) protect zebrafish hair cells *in vivo* from neomycin (6.25 μM, 1 h). Protection of zebrafish hair cells against gentamicin (10 μM, 6 h) was provided by ≥25 μM dTC or ≥12.5 μM berbamine. Hair cells in mouse cochlear cultures are protected from longer-term exposure to gentamicin (5 μM, 48 h) by 20 μM berbamine or 25 μM dTC. Berbamine is, however, highly toxic to mouse cochlear hair cells at higher concentrations (≥30 μM) whilst dTC is not. The absence of toxicity in the zebrafish assays prompts caution in extrapolating results from zebrafish neuromasts to mammalian cochlear hair cells. MET current recordings from mouse outer hair cells (OHCs) show that both compounds are permeant open-channel blockers, rapidly and reversibly blocking the MET channel with half-blocking concentrations of 2.2 μM (dTC) and 2.8 μM (berbamine) in the presence of 1.3 mM Ca^2+^ at −104 mV. Berbamine, but not dTC, also blocks the hair cell's basolateral K^+^ current, I_K,neo_, and modeling studies indicate that berbamine permeates the MET channel more readily than dTC. These studies reveal key properties of MET-channel blockers required for the future design of successful otoprotectants.

## Introduction

Aminoglycoside antibiotics are prescribed worldwide as an effective treatment for serious and life-threatening conditions including tuberculosis, sepsis, neonatal infections, and those associated with cystic fibrosis (Rizzi and Hirose, 2007; Durante-Mangoni et al., 2009). The potency of these drugs against such infections ensures their continued use despite the knowledge that they are both nephro- and ototoxic (Forge and Schacht, 2000). Whilst kidney damage is reversible, a degree of permanent hearing loss is found in around 20–30% of patients treated with these antibiotics (Rizzi and Hirose, 2007; Schacht et al., 2012). The hearing loss is the result of damage caused to the sensory hair cells in the inner ear, an organ in which the aminoglycosides are found to selectively accumulate, with the basal, high-frequency outer hair cells (OHCs) being those that are predominantly affected (Forge and Schacht, 2000; Nakashima et al., 2000).

The main route of aminoglycoside entry into the hair cells is via their mechano-electrical transducer (MET) channels, large non-selective cation channels located at the tips of the hair cells' stereocilia (Marcotti et al., 2005; Alharazneh et al., 2011; Vu et al., 2013). Evidence for the molecular identity of the MET channel is increasingly suggesting that the transmembrane channel-like (TMC) family proteins, TMC1, and TMC2, are prime candidates (Kawashima et al., 2011, 2015; Pan et al., 2013; Kurima et al., 2015; Corey and Holt, 2016; Fettiplace, 2016). Once inside the cells the aminoglycosides disrupt various pathways and organelles, resulting in the activation of multiple signaling cascades including those involving the caspases (Forge and Li, 2000; Matsui et al., 2002, 2004; Owens et al., 2007). In the absence of alternative antibiotics of similar efficacy, identifying methods to protect the hair cells from this damage is crucial. Although one approach is to interrupt the intracellular pathways this may prove complex as, for example, the two closely-related aminoglycosides neomycin and gentamicin have been found to activate distinct cell-death pathways in zebrafish lateral line hair cells (Owens et al., 2009; Coffin et al., 2013a,b). Arguably, a more effective and universal method would be to administer compounds that block the MET channels and prevent aminoglycoside entry into the cells.

d-Tubocurarine (dTC), historically known as the main active component of the arrow poison, curare, is a naturally occurring alkaloid obtained from the bark of the South American plant *Chondrodendron tomentosum* (Perotti, 1977). It is a nicotinic antagonist that has been shown to block the acetylcholine receptor response in mature guinea-pig OHCs (Housley and Ashmore, 1991; Eróstegui et al., 1994) as well as the MET channels in neonatal mouse cochlear OHCs (Glowatzki et al., 1997). A study into the pore of the MET channels in turtle auditory hair cells reported that curare acts as a non-permeant blocker of these channels (Farris et al., 2004), making this an interesting molecule to investigate for potential otoprotective properties. The co-application of 1 mM curare was shown to significantly reduce the loading of 3 μM Texas Red conjugated gentamicin (GTTR) into rat cochlear inner hair cells (IHCs) and OHCs (Alharazneh et al., 2011) suggesting a competitive block of the pore, and the presence of 1 mM curare during the exposure of rat cochlear cultures to 0.1 mM gentamicin prevented hair-cell death from occurring during a subsequent 48-h antibiotic-free period (Alharazneh et al., 2011).

Looking for other potential MET channel blockers, we identified berbamine as having a very similar chemical structure to dTC. Berbamine is a naturally occurring alkaloid that is present in a number of plant species within the *Berberidaceae* family (Rahmatullah et al., 2014). It has been used in Eastern medicine for centuries to treat inflammation and related conditions such as rheumatoid arthritis and is still of interest to date for its potential anti-cancer properties (Ji et al., 2009; Meng et al., 2013; Rahmatullah et al., 2014; Zhao et al., 2016). Studies on zebrafish lateral line hair cells have revealed that 25 μM berbamine can protect these cells from the damage caused by 50–400 μM of either neomycin or gentamicin (Kruger et al., 2016). Furthermore, these authors found that berbamine blocked the loading of both GTTR and FM1-43, a styryl dye that acts as a permeant blocker of the hair cells' MET channels (Gale et al., 2001), leading Kruger et al. (2016) to conclude that berbamine is providing protection by competitively blocking the MET channels.

The zebrafish lateral line system is a useful and effective model for initial screening, with the hair cells being both structurally and functionally similar to mammalian inner ear hair cells and externally located, making them easily accessible for pharmacological studies. A degree of caution is however required as to date only three of the compounds that have been identified to protect lateral line hair cells have also been shown to protect mammalian inner ear hair cells (PROTO1, tacrine, and phenoxybenzamine; Owens et al., 2008; Ou et al., 2009; Majumder et al., 2017). We therefore sought to ascertain if berbamine would protect mammalian hair cells from the toxic side effects of aminoglycoside antibiotics and, likewise, if dTC would protect zebrafish lateral line hair cells. Furthermore, we fully characterized how dTC blocks the MET channel in mammalian OHCs and whether berbamine, like dTC, acts in a similar fashion or otherwise. Comparisons between the two structurally related molecules will assist in understanding the features/characteristics of a compound that are required to provide optimum otoprotection.

## Materials and methods

### Zebrafish husbandry and embryo generation

Zebrafish embryos were obtained from sibling crosses of adult AB fish. Embryos were staged following standard protocols (Kimmel et al., 1995; Westerfield, 2000) and raised at 28.5°C in E3 medium (1 mM NaCl, 0.17 mM KCl, 0.33 mM MgSO_4_, 0.33 mM CaCl_2_).

### Zebrafish protection assay

AB larvae (4 days post fertilization, dpf) were pre-incubated with 3 μM of Yo-Pro-1 (Molecular Probes Y3603) for 30 min to label the hair cells, washed and pipetted into 96-well plates (three larvae per well). Larvae were co-incubated with serial dilutions of test compound (dTC or berbamine) ranging from 200 to 1.55 μM with 6.25 μM neomycin sulfate (Sigma N1876) for 1 h, or with 10 μM gentamicin sulfate (Sigma G3632) for 6 h. Plates were screened on a Zeiss IM35 inverted microscope with a 16X objective. Trunk neuromasts 3–9 were viewed for qualitative assessment of damage. Images of trunk neuromast 4 of all three larvae in each well were taken on a Nikon D5000 at 40X magnification and individual cells within the neuromast counted for quantitative analysis. Experiments were repeated at least four times.

### Mouse cochlear culture preparation

Cochlear cultures were prepared from wild-type CD-1 mice as previously described by Russell and Richardson (1987). In brief, postnatal day 2 (P2) pups of either sex were killed by cervical dislocation following Home Office guidelines. Decapitated heads were surface sterilized by three 1-min washes in 80% ethanol. Sagittal incisions were made down the midline of the head and cochleae were removed. Subsequent dissections were performed in Hanks' Balanced Salt Solution (HBSS; Thermo Shandon 14025050) buffered with 10 mM Hepes (Sigma H0887) (HBHBSS). Cochleae were removed from the bony labyrinth and explanted onto collagen-coated (Corning 354236) coverslips in cochlear culture medium (93% DMEM-F12, 7% fetal bovine serum and 10 μg.ml^−1^ ampicillin). The coverslips complete with cochleae were then sealed in Maximow slide assemblies and the cultures were left to grow and adhere to the collagen in a 37°C incubator before use.

### Mouse cochlear culture protection assay

Following 24 h incubation coverslips with adherent cochleae were removed from the Maximow slide assemblies, placed in 35 mm petri dishes (Greiner Bio-One 627161) and incubated for 48 h in the presence of 1 ml of cochlear culture medium that had been diluted with DMEM-F12 to reduce the serum concentration to 1.4%, together with 5 μM gentamicin (Sigma G3632) and varying concentrations of berbamine or dTC. Following 48-h incubation, cultures were washed in phosphate buffered saline (PBS), fixed in 3.7% formaldehyde (Sigma F1635) in 0.1 M sodium phosphate buffer pH 7.4, and stained with TRITC-phalloidin (Sigma P1951). Cultures were mounted on glass slides with Vectashield (Vector Laboratories H-1000) and imaged using a Zeiss Axioplan2 microscope. Images were obtained from the middle of the basal coil at a position ~20% along the length of the cochlea, measured from the basal tip. For quantification, the OHCs in these images were counted and averaged across a number of experiments. Image width was 220 μm and was aligned along the length of the cochlea.

### Mouse cochlear culture electrophysiology

Recordings were made from OHCs in organotypic cultures that had been maintained for 1–3 days *in vitro*. The organotypic cultures were transferred to the microscope chamber on their collagen-coated coverslips and the chamber was continuously perfused with an extracellular solution containing (in mM): 135 NaCl, 5.8 KCl, 1.3 CaCl_2_, 0.9 MgCl_2_, 0.7 NaH_2_PO_4_, 5.6 D-glucose, 10 HEPES-NaOH, 2 sodium pyruvate. MEM amino acids solution (50X) and MEM vitamins solution (100X) were added to a final concentration of 1X from concentrates (Fisher Scientific). The pH was adjusted to 7.48 with 1 molar NaOH (osmolality ~308 mOsmol kg^−1^). The organs of Corti were observed with an upright microscope (Olympus) with Nomarski differential interference contrast optics (40X water-immersion objective). Whole-cell patch-clamp recordings were obtained from basal-coil OHCs at room temperature (20–23°C) using an Optopatch (Cairn Research) patch-clamp amplifier. For MET current recordings patch pipettes (2.5–3.0 MΩ) contained the following (in mM): 137 CsCl, 2.5 MgCl_2_, 1 EGTA-CsOH, 2.5 Na_2_ATP, 10 sodium phosphocreatine, 5 HEPES-CsOH; pH adjusted to 7.3 with CsOH (osmolality ~295 mOsmol kg^−1^). For recording basolateral potassium currents patch pipettes contained (in mM): 131 KCl, 3 MgCl_2_, 5 Na_2_ATP, 1 EGTA-KOH, 5 HEPES, 10 sodium phosphocreatine, pH adjusted to 7.28 with KOH (osmolality ~295 mOsmol kg^−1^). Patch pipettes were coated with surf wax (Mr. Zogs SexWax) to minimize the fast capacitance transient across the wall of the patch pipette. MET currents were elicited by stimulating the OHC hair bundles using a fluid jet from a pipette (tip diameter 8–10 μm) driven by a piezoelectric disc (Kros et al., 1992; Marcotti et al., 2005). Mechanical stimuli (filtered at 1.0 kHz, 8-pole Bessel) were applied as 45 Hz sinusoids or, to quantify kinetics of block, voltage steps, with driver voltage amplitudes of ±40 V, sufficient to elicit large, saturating MET currents. Currents were acquired using pClamp (Molecular Devices) software and stored on a computer for off-line analysis. To look at extracellular block the compounds were locally superfused onto the OHCs at concentrations ranging from 300 nM to 100 μM in a solution containing (in mM): 145 NaCl, 5.8 KCl, 1.3 CaCl_2_, 0.9 MgCl_2_, 0.7 NaH_2_PO_4_, 5.6 glucose, 10 HEPES-NaOH, 2 sodium pyruvate. The pH was adjusted to 7.48 with NaOH (osmolality ~305 mOsmol kg^−1^). This solution, without any compound, was superfused as a control solution before and after the application of each compound. A modest negative pressure applied to the tip of the fluid jet pipette resulted in the compound-containing solution being sucked into the pipette during superfusion. This prevented mixing and dilution of the compound with bath solution during fluid jet stimulation. To look at intracellular block the compounds were included in the patch-pipette solution at concentrations ranging from 100 μM to 1 mM. MET currents obtained at the beginning of each recording were used as controls, by which time the compounds would not yet have diffused into the cells.

MET current size was determined by measuring the difference between the minimum current during the inhibitory phase of the sinewave and the current ~6 ms after the onset of the excitatory phase of the sinewave, a time point at which the current would have reached near steady state. The current sizes were averaged for each 22 ms phase of the sinewave, omitting the first cycle. Basolateral currents were determined by measuring the steady-state current toward the end of the voltage-step. Series resistance compensation was applied (50–80%) and the average residual series resistance was calculated to be 1.73 ± 0.10 MΩ (*n* = 78). The average maximum MET current size was 1.54 ± 0.08 nA (*n* = 66), and the average maximum basolateral current was 2.09 ± 0.11 nA (*n* = 12). This would result in a maximum voltage drop across the residual series resistance of 2.7 and 3.6 mV respectively, considered sufficiently small to not require any correction to quoted voltage values. All voltages reported include a −4 mV correction for the liquid junction potential between extra- and intra-cellular solutions.

### Two-barrier one binding-site model of permeant block of the MET channel

The model used is a modification of that used for describing permeation and block by dihydrostreptomycin (DHS; Marcotti et al., 2005), to allow for values of the Hill coefficient (a measure of the degree of cooperativity of the binding process of blocker molecules to the binding site) that are greater or less than unity (van Netten and Kros, 2007). Assuming that the fraction of unblocked transducer channels is indicated by *C*, the fraction of blocked channels by *CB*, the extra- and intracellular blocker by *B*_*o*_ and *B*_*i*_, the Hill coefficient by *n*_H_, and using the forward (*k*_1_*, k*_2_) and reverse (*k*_−1_*, k*_−2_) rate constants, the reaction equation is given by:

(1) $$\begin{array}{r} C+n_{H}B_{0}\underset{k_{-1}}{\overset{k_{1}}{\Leftrightarrow }}CB_{n_{H}}\underset{k_{-2}}{\overset{k_{2}}{\Leftrightarrow }}C+n_{H}B_{i}. \end{array}$$

Introducing the time variable *t*, the dynamics of the two fractions are denoted by *C*(*t*) and *CB*(*t*) = 1− *C*(*t*). The rate of change of *C*(*t*) is dependent on the four rate constants *k*_1_, *k*_−1_, *k*_2_, *k*_−2_ of the transitions across the barriers and the intra- and extracellular blocker concentrations, [*B*_*i*_] and [*B*_*o*_]. We assume that the intracellular compound concentration *B*_*i*_ is small and it is therefore set to zero ([*B*_*i*_ = 0]).

The voltage across the MET channel is assumed to vary with a fixed gradient so that it linearly changes the free energy across the membrane, effectively tilting the overall free energy profile in proportion to the membrane potential *V*. The maxima of the free energy related to the barriers are defined as *E*_1_ and *E*_2_ and the minimum free energy related to the binding site as *E*_b_, with respect to *V* = 0. We further assume that the two barriers are located at both sides of the membrane so that their fractional positions across the membrane are δ_1_ = 0 (outside), and δ_2_ = 1 (inside). This is a simplification of, but otherwise similar to, that used in a previous study of block by DHS, where the barriers were positioned just inside the field (Marcotti et al., 2005).

### Block of GTTR loading in mouse cochlear cultures

Cochlear cultures on the collagen-coated coverslips were transferred from the Maximow slide assemblies into a viewing chamber in which they were incubated for 5 min in HBHBSS together with 1% DMSO and 100 μM of compound (dTC or berbamine) or 1% DMSO vehicle alone. GTTR was then added to a final concentration of 0.2 μM and left for 10 min. The cultures were washed three times and imaged using a 60X dipping lens on a Zeiss Axioplan2 microscope. Fluorescence intensity values were obtained from ten cells for each condition from images captured at a time point 27 min from the start of the experiment. Experiments for each condition were repeated three times (30 cells total) and the intensity values averaged. Images were obtained from a region that was ~800 μm from the basal tip of the cochlea.

### Compound analysis

Structures of dTC and berbamine were prepared, energy minimized (using MMFF94x forcefield) and flexibly aligned using Molecular Operating Environment (MOE) 2015.10. pKa was calculated using MarvinSketch 16.8.15.0, ChemAxon (https://www.chemaxon.com).

### Statistical analysis

Values of half-blocking concentration (*K*_*D*_) and Hill coefficient (*n*_H_) determined from fitting dose-response curves were tested for significant differences using 95% confidence intervals (CI). This is equivalent to *p* < 0.05 being the criterion for statistical significance. Multiple comparisons were made using 1-way ANOVA with Dunnett (cell counts) or Tukey (GTTR fluorescence) post tests. Compounds were considered fully protective if the cell counts differed significantly from the cell counts in the presence of aminoglycoside antibiotic (neomycin or gentamicin) alone, but not from the cell counts in the control medium. Compounds were considered partially protective if cell counts differed significantly from both aminoglycoside and control media. Means are quoted and shown in Figures ± SEM. Level of statistical significance is shown in Figures as follows: ^*^p < 0.05; ^**^p < 0.01; ^***^p < 0.001.

### Study approval

Animals were raised following Home Office guidelines. All experiments were performed in accordance with the Home Office Animals (Scientific Procedures) Act 1986 and approved by the University of Sussex Animal Welfare and Ethical Review Board.

## Results

### d-tubocurarine and berbamine protect zebrafish lateral line hair cells from aminoglycoside damage

**Figure 1:**
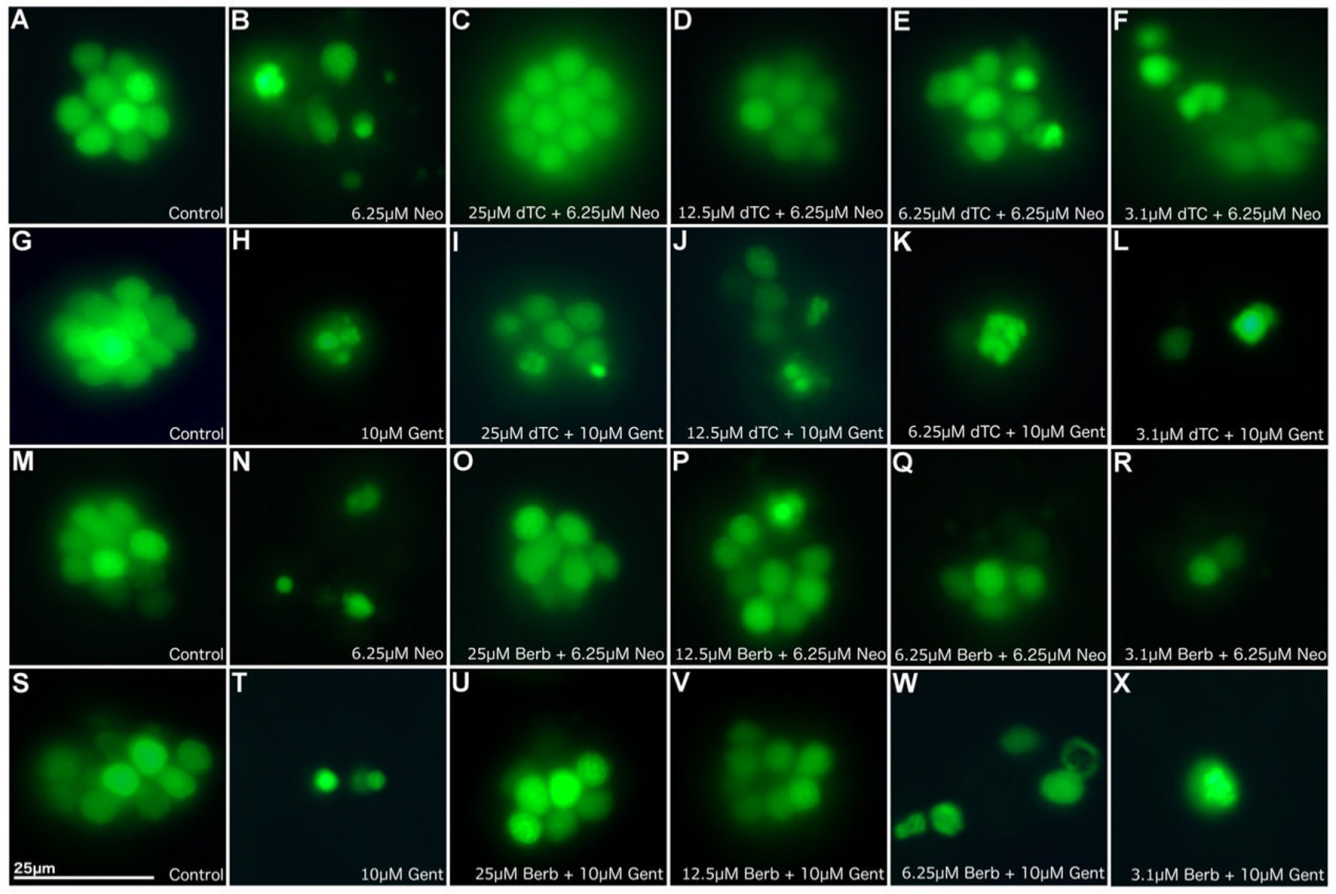
d-Tubocurarine and berbamine protect lateral line hair cells in zebrafish from gentamicin and neomycin induced damage. Zebrafish were treated with: **(A,G,M,S)** E3 alone **(B)** 6.25 μM neomycin, or **(C–F)** 25, 12.5, 6.25, or 3.1 μM of dTC plus 6.25 μM neomycin, **(H)** 10 μM gentamicin, **(I–L)** 25, 12.5, 6.25, or 3.1 μM of dTC plus 10 μM gentamicin, **(N)** 6.25 μM neomycin, **(O–R)** 25, 12.5, 6.25, or 3.1 μM of berbamine plus 6.25 μM neomycin, **(T)** 10 μM gentamicin, **(U–X)** 25, 12.5, 6.25, or 3.1 μM of berbamine plus 10 μM gentamicin. Neuromasts were pre-stained with 3 μM Yo-Pro1. *n* > 3 independent experiments with at least 3 fish per well. Images were obtained with a 40X objective. Scale bar = 25 μm.

**Figure 2:**
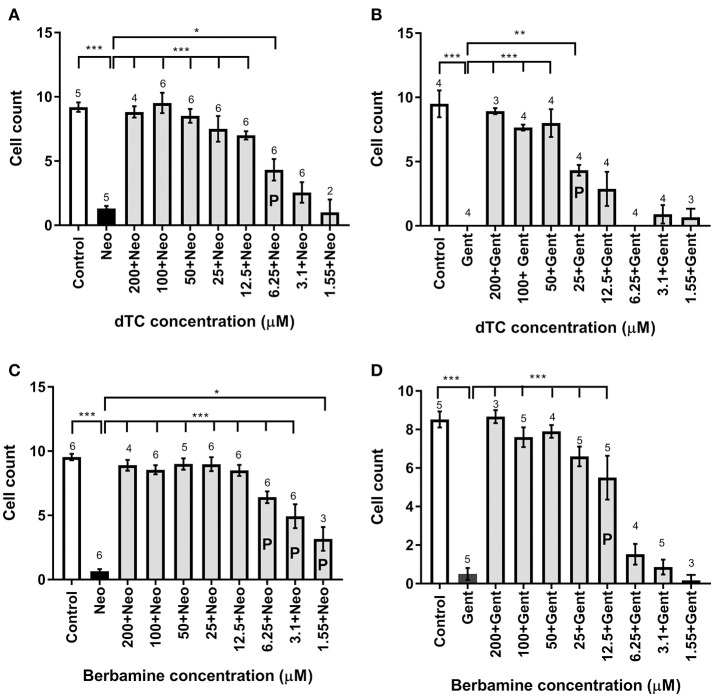
Cell count analysis for lateral line hair cell protection against gentamicin and neomycin by d-tubocurarine and berbamine. **(A–D)** A range of concentrations (1.55–200 μM) of either dTC or berbamine with either neomycin or gentamicin are compared to neomycin or gentamicin alone. Number of hair cells were counted in trunk neuromast 4. **(A)** dTC against neomycin, **(B)** dTC against gentamicin, **(C)** berbamine against neomycin and **(D)** berbamine against gentamicin. Cells counts that are significantly different from aminoglycoside alone are indicated above the bars, with the number of stars representing the level of significance. The letter **P** in the bars indicates concentrations that were deemed partially protective, because cell counts were also significantly different from the control (A: ^***^; **B**: ^***^; **C**: ^***^; **D**: ^**^). Numbers above the bars indicate experimental replicates, with cell numbers in three neuromasts counted and averaged for each replicate. ^*^p < 0.05; ^**^p < 0.01; ^***^p < 0.001.

In order to assess the protective capabilities of dTC and berbamine against aminoglycoside damage, a protection assay was performed using hair cells in the lateral line organs of zebrafish larvae at 4 dpf. This time point ensures the reliable loading of Yo-Pro 1 (Santos et al., 2006; Kindt et al., 2012). dTC was found to fully protect against damage induced by 6.25 μM neomycin in zebrafish larvae at concentrations ≥12.5 μM, with partial protection at 6.25 μM (Figures 1A–F, 2A). Full protection against damage induced by 10 μM gentamicin was only observed at concentrations ≥50 μM dTC, with partial protection at 25 μM (Figures 1G–L, 2B). Berbamine protected at somewhat lower concentrations compared to dTC. It fully protected against neomycin-induced neuromast damage in zebrafish larvae at concentrations ≥12.5 μM, with partial protection even down to 1.55 μM, the lowest concentration tested (Figures 1M–R, 2C). Berbamine offered full protection against gentamicin damage at 25 μM and above, with partial protection at 12.5 μM (Figures 1S–X, 2D). No signs of toxicity due to either compound were observed at the highest concentration tested (200 μM).

### d-tubocurarine and berbamine protect mouse cochlear hair cells from aminoglycoside damage, but berbamine is toxic at higher concentrations

**Figure 3:**
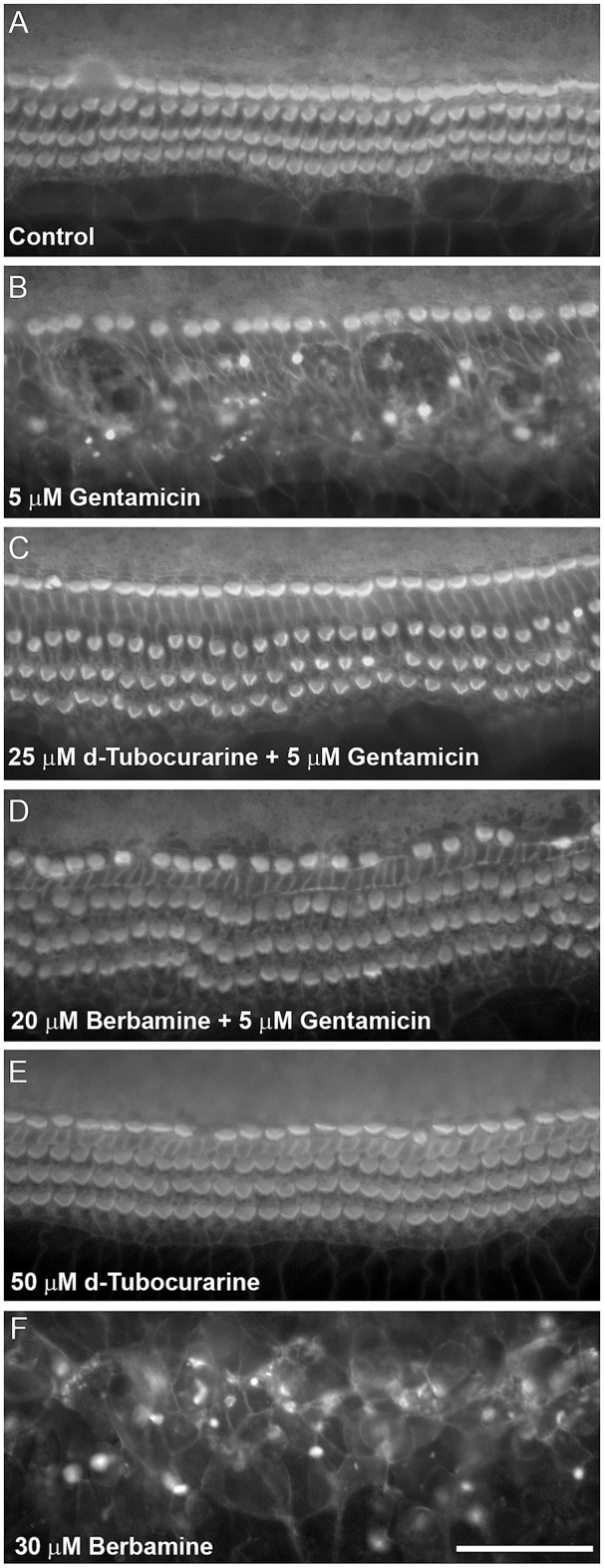
d-Tubocurarine and berbamine protect cochlear OHCs from gentamicin damage. Protection assay using cultures prepared from CD-1 mice at postnatal day 2 (P2). Cultures were stained with TRITC-phalloidin at the end of the experiment allowing visualization of the actin-rich stereocilia. **(A)** Control culture incubated for 48 h in the presence of medium alone. The sensory hair cell loss shown in **(B)** caused by 48 h incubation with 5 μM gentamicin can be prevented by co-incubation with 25 μM dTC **(C)** or 20 μM berbamine **(D)**. Cells incubated in 50 μM dTC in the absence of gentamicin were not damaged **(E)**, whereas 30 μM berbamine resulted in widespread loss of IHCs, OHCs and other cell types **(F)**. Scale bar = 50 μm.

**Figure 4:**
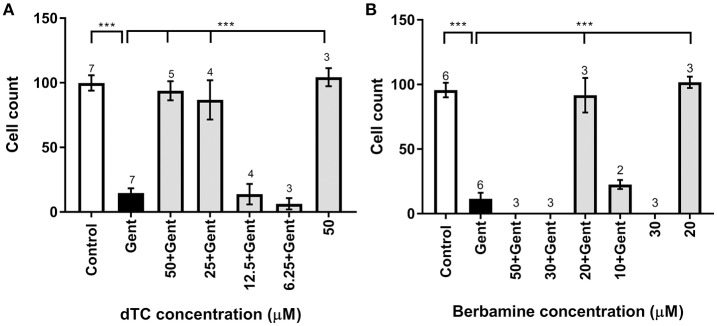
Quantification of survival of OHCs exposed to gentamicin and otoprotective compounds. **(A,B)** Bar charts showing average numbers of OHCs remaining after 48 h in medium alone (control), in medium containing 5 μM gentamicin, in 5 μM gentamicin plus different concentrations of compounds, and in medium containing the compound alone. Number of OHCs were counted in a 220 μm wide field. Significant protection occurs at dTC concentrations of 25 μM and above **(A)** and for 20 μM berbamine **(B)**. While 50 μM dTC alone does not cause OHC damage, OHCs are obliterated at ≥30 μM berbamine, with or without gentamicin. Numbers above the bars indicate experimental replicates. ^***^p < 0.001.

dTC and berbamine were subsequently tested in mouse cochlear cultures to see if they would protect mammalian cochlear hair cells from the damage induced by exposure to 5 μM gentamicin for 48 h (Figures 3A–D). Such treatment results in the nearly complete loss of OHCs from the basal 35% of the cochlea, but has little effect if any on the survival of IHCs. dTC and berbamine were found to be protective at minimum concentrations of 25 μM and 20 μM, respectively, as quantified in Figures 4A,B, which show counts of OHCs in a 220 μm wide segment of the basal coil of the cochlea. As a further criterion to assess the suitability of a compound for use as an otoprotectant we examined the hair-bundle morphology. No hair-bundle damage was observed during exposure to either compound.

To determine if either compound had any adverse effects on hair cells in the absence of gentamicin, they were tested alone at a higher concentration of 50 μM. Berbamine was found to be generally cytotoxic and killed both the IHCs and OHCs as well as other cell types in the entire organ of Corti, whereas dTC had no adverse effects on hair cells or hair-bundle morphology at this concentration (Figures 3E, 4A). Berbamine was tested alone at the lower concentrations of 20 and 30 μM and found to be equally toxic at 30 μM as it was at 50 μM (no IHCs or OHCs could be identified) but not at 20 μM, the concentration at which it showed protection against 5 uM gentamicin (Figures 3F, 4B). When 30 μM berbamine was tested together with 5 μM gentamicin, the same cytotoxic effect was observed as with 30 μM berbamine alone. While berbamine can thus protect from gentamicin toxicity, albeit over a narrow concentration range, we saw no evidence that gentamicin could protect from berbamine cytotoxicity.

### d-tubocurarine and berbamine block MET channel currents in mouse cochlear hair cells

**Figure 5:**
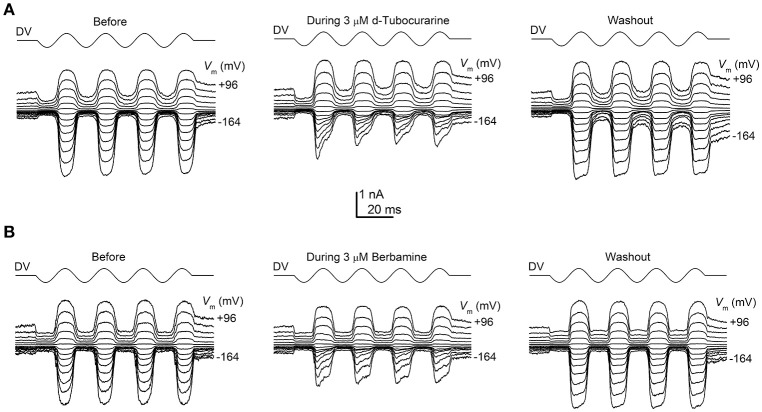
Extracellular d-tubocurarine and berbamine block the OHC MET channel. **(A,B)** MET currents recorded from P2+2 OHCs before, during and after superfusion with a solution containing 3 μM dTC **(A)** or 3 μM berbamine **(B)**. Both compounds can be seen to predominantly block the MET currents at hyperpolarized potentials with the block fully reversible as seen from the washouts. Membrane potentials were stepped between −164 mV and +96 mV and MET currents were recorded in response to a sinewave delivered by a fluid jet (45 Hz sinusoid, ±40 V driver voltage, DV) shown above each trace. The capacitances of the cells were 6.9 pF **(A)** and 7.8 pF **(B)**.

To determine whether the protection observed for both compounds may be the result of a direct interaction with the hair cells' MET channels, thereby reducing or preventing aminoglycoside entry, we examined the effect of dTC and berbamine on the MET currents in basal-coil OHCs, the cells that are predominantly affected by aminoglycoside ototoxicity. Although dTC has previously been described as a MET channel blocker (Glowatzki et al., 1997; Farris et al., 2004), no such study has been carried out with berbamine. MET currents were recorded at membrane potentials ranging from −164 to +96 mV before, during and after superfusion with dTC or berbamine at concentrations ranging from 300 nM to 100 μM (dTC), or 1 to 30 μM (berbamine). During exposure to either dTC or berbamine, a reduction in the current sizes was seen when the cells were stepped to hyperpolarized potentials. This reduction was less pronounced at depolarized potentials, with the level of the reduction being dependent upon the concentration of the compound. Examples of MET current block by dTC and berbamine at a concentration of 3 μM are shown in Figures 5A,B. Note that currents before and after application of the compounds appear as rectified versions of the sinewave stimulus. During superfusion of the compounds the currents look similar at positive potentials, but at negative potentials the MET currents can be seen to decline rapidly on each cycle after an initial inward current peak. This is suggestive of an open-channel blocking mechanism, where the blocker can only interact with the open channel. The block of the channel and subsequent washout were both rapid, with the currents making a full recovery upon re-exposure to the control superfusion solution, indicating that the block by both dTC and berbamine is completely reversible (Figures 5A,B).

**Figure 6:**
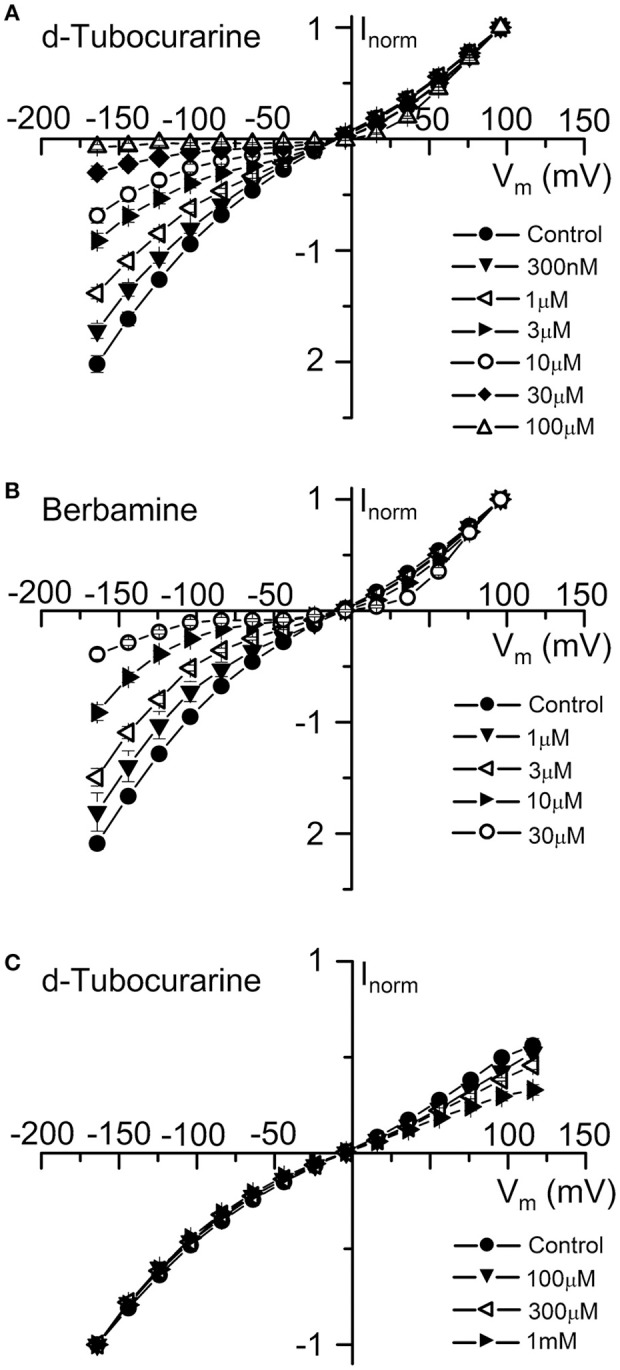
Normalized current-voltage curves reveal the voltage-dependence of d-tubocurarine and berbamine MET channel block. **(A,B)** Average normalized current-voltage curves for the peak MET currents recorded before and during extracellular superfusion with 300 nM to 100 μM dTC **(A)**, or 1 to 30 μM extracellular berbamine **(B)**. Currents were normalized to the peak current measured at +96 mV. For the two compounds, block increases with both increasing hyperpolarization and compound concentration. Inclusion of 0.1 to 1 mM dTC in the patch-pipette resulted in a block of the MET currents from the intracellular side at depolarized potentials **(C)**. Currents in **(C)** were normalized to the peak current measured at −164 mV. Numbers of cells and peak current **(A)** Control: 43, 0.92 ± 0.05 nA; 300 nM: 5, 1.01 ± 0.14 nA; 1 μM: 6, 1.00 ± 0.09 nA; 3 μM: 9, 0.89 ± 0.10 nA; 10 μM: 5, 0.75 ± 0.09 nA; 30 μM: 13, 0.78 ± 0.10 nA; 100 μM: 5, 0.32 ± 0.03 nA **(B)** Control: 37, 0.65 ± 0.07 nA; 1 μM: 6, 0.76 ± 0.19 nA; 3 μM: 14, 0.51 ± 0.10 nA; 10 μM: 8, 0.54 ± 0.11 nA; 30 μM: 9, 0.38 ± 0.11 nA **(C)** Control: 10, −1.86 ± 0.13 nA; 100 μM: 3, −1.84 ± 0.31 nA; 300 μM: 2, −1.95 ± 0.12 nA; 1 mM: 5, −1.40 ± 0.13 nA.

Average normalized current-voltage curves show the extracellular block by dTC and berbamine at a range of concentrations (Figures 6A,B). These curves clearly demonstrate both the increase in the block with increasing compound concentration and the voltage dependence of the block, with minimal block at the depolarized potentials and stronger block at the hyperpolarized potentials.

To examine whether either compound has an effect on the MET currents when applied from the intracellular side of the channel, dTC and berbamine were included in the intracellular patch pipette solution at varying concentrations to enable their entry into the cells. Transducer currents were recorded in the same way as for the extracellular experiments, but omitting any local superfusion of the cells. Intracellular berbamine at concentrations of 100 and 300 μM was found to have no effect on the size of the MET currents (data not shown). A higher concentration of 1 mM resulted in the rapid loss of the cells and the inability to maintain whole-cell recordings in every cell tested. Conversely, intracellular dTC, at concentrations ranging from 100 μM to 1 mM, was found to reduce the current size when cells were depolarized but not when they were hyperpolarized, indicating a block of the channel from the intracellular side. Average normalized current-voltage curves showing this intracellular block are shown (Figure 6C). Again, these curves clearly demonstrate the voltage dependence of the block and the increase in block with increasing compound concentration.

**Figure 7:**
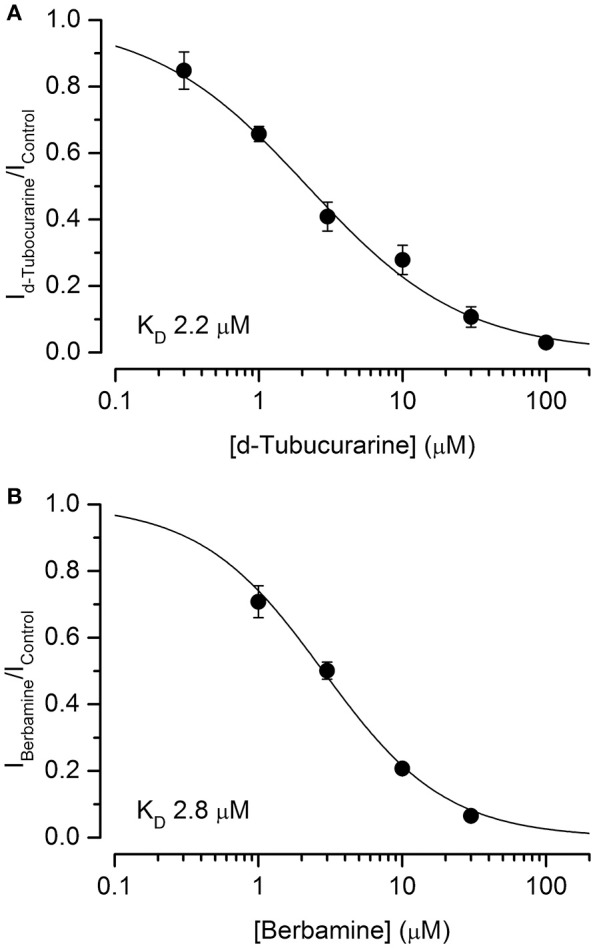
d-Tubocurarine and berbamine block MET channels with similar half-blocking concentrations. **(A,B)** Dose-response curves of MET channel block by dTC **(A)** and berbamine **(B)** derived from currents recorded at −104 mV and fit with Equation (2). dTC: *K*_*D*_ 2.2 μM, Hill coefficient 0.80; berbamine: *K*_*D*_ 2.8 μM, Hill coefficient 1.02. From 5 to 14 cells were used for each data set.

Dose-response curves for the extracellular block of the MET channels by dTC and berbamine were generated (Figures 7A,B). These curves were derived from the currents measured at −104 mV, near the membrane potential at which the block was seen to be the strongest, and fitted with the equation:

(2) $$\begin{array}{r} \frac{I}{I_{C}}=\frac{1}{1+{\left(\frac{\left[B\right]}{K_{D}}\right)}^{n_{H}}}\; \end{array}$$

where *I*_*C*_ is the control current in the absence of the compound, [*B*] is the concentration of the blocking compound, *K*_*D*_ is the half-blocking concentration and *n*_*H*_ is the Hill coefficient. From these curves, the *K*_*D*_ for dTC was found to be 2.2 μM (95% CI 1.7 μM to 2.7 μM), a value very similar to the previously reported value for neonatal mouse OHC MET channel block of 2.3 μM (Glowatzki et al., 1997). The *K*_*D*_ for berbamine was found to be 2.8 μM (95% CI 2.4–3.2 μM), similar to the styryl dye FM1-43 (2.4 μM at −104 mV: Gale et al., 2001) and not significantly different from dTC. Both dTC and berbamine have a higher affinity for the MET channel than DHS, which has a reported *K*_*D*_ of 10 μM (Marcotti et al., 2005). The Hill coefficients calculated from the dose-response curve fits were 1.02 for berbamine and 0.80 for dTC, the latter a value that is significantly smaller than one (*p* < 0.05). These values suggest that, in the case of berbamine, a single molecule interacts with and blocks the channel whereas for dTC two molecules are involved, showing negative cooperativity (Wyman and Gill, 1990).

A dose-response curve for the intracellular block of the MET channels by dTC was generated, derived from the currents measured at +96 mV and fitted with Equation (2) (not shown). From this curve dTC was found to block the intracellular side of the channel with a *K*_*D*_ of 880 μM (95% CI 700–1,070 μM) and a Hill coefficient of 0.92, values similar to those found for the intracellular block of the MET channel by DHS (Marcotti et al., 2005; Corns et al., 2016). These values show that the affinity of dTC for the MET channel is greatly reduced when blocking from the intracellular side as opposed to the extracellular side.

**Figure 8:**
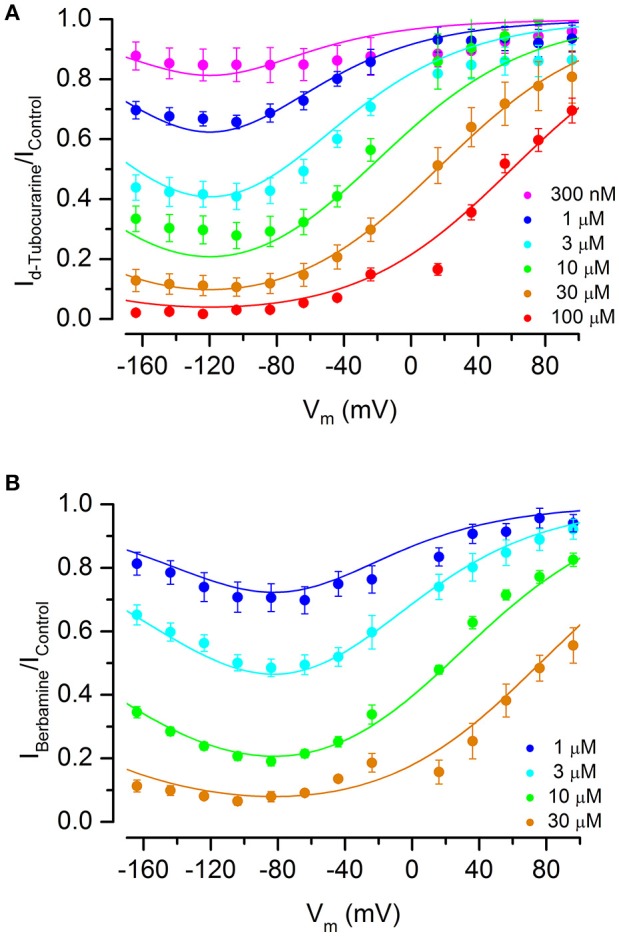
Fractional block of the MET currents by d-tubocurarine and berbamine shows that both are permeant blockers. **(A,B)** Fraction of the MET current remaining during dTC **(A)** or berbamine **(B)** superfusion relative to the control current at the same membrane potential. Concentrations ranged from 300 nM to 100 μM (dTC) or 1 to 30 μM (berbamine). Data are fitted with a two-barrier, one binding-site model (see Section Materials and Methods). A release of the block at extreme hyperpolarized potentials indicates that both compounds can enter the cells. Maximum current block is seen at −118 mV (dTC) and −94 mV (berbamine). Fitted parameters were for dTC ΔE 4.96 kT; E_b_ −8.67 kT; z 1.09; δ_b_ 0.52, and for berbamine ΔE 2.68 kT; E_b_ −12.0 kT; z 0.94; δ_b_ 0.57. Number of cells **(A)** 300 nM: 5, 1 μM: 6, 3 μM: 9, 10 μM: 5, 30 μM: 13, 100 μM: 5 **(B)** 1 μM: 6, 3 μM: 14, 10 μM: 8, 30 μM: 9.

Fractional block curves for both dTC and berbamine at all concentrations tested were plotted (Figures 8A,B). The data were fitted with a two-barrier, one binding-site (2B1BS) model similar to that previously used to describe the block of the MET currents by DHS (Marcotti et al., 2005) but adapted to allow for Hill coefficients different from one (van Netten and Kros, 2007; see Section Materials and Methods). For plotting the fractional block, the half-blocking concentration *K*_*D*_ in Equation (2) above becomes voltage-dependent as follows:

(3) $$\begin{array}{l} \;K_{D}={\left[K_{1}(V)\right]}^{\frac{1}{nH}}\text{ with}\\ K_{1}(V)=\exp\;\left(\frac{E_{b}}{kT}+\frac{\delta_{b}V}{V_{s}}\right)\cdot \left(1+\exp\;\left(-\frac{\Delta E}{kT}-\frac{V}{V_{s}}\right)\right), \end{array}$$

where Δ*E* = *E*_2_ − *E*_1_, the slope factor: $V_{s}=\frac{kT}{ze_{0}}$, is the ratio of thermal energy (*kT*, i.e., Boltzmann's constant multiplied by absolute temperature) and effective charge of the blocker molecule (*ze*_0_, i.e., valence multiplied by elementary charge) and δ_*b*_ is the relative electrical distance of the binding site along the membrane.

The data and fitted curves show that block is voltage-dependent for both compounds, with block being released strongly at positive potentials but also, to a lesser extent, at extreme negative potentials—the latter indicative of permeant block in which the compounds enter the cell with a sufficiently strong electrical driving force. This release at hyperpolarized potentials was more pronounced for berbamine than for dTC. The membrane potential of maximum block was −118 mV for dTC and −94 mV for berbamine.

**Figure 9:**
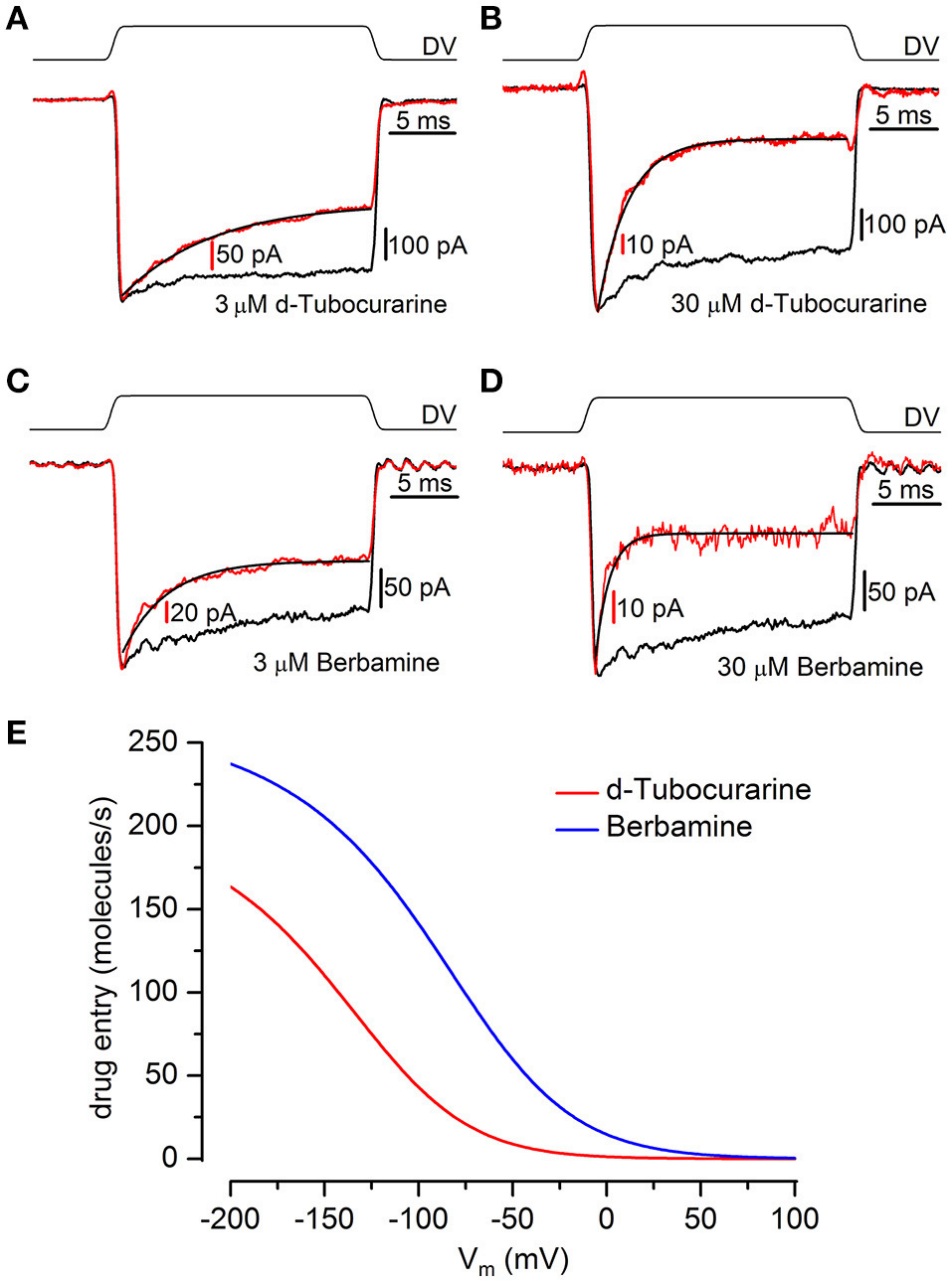
Kinetics of MET channel block reveal d-tubocurarine and berbamine act as open channel blockers. **(A–D)** Transducer currents were recorded from OHCs in response to a mechanical step delivered by a fluid-jet (±40 V driver voltage, DV), shown above each trace [**(A,B)** P2+2; **(C,D)** P2+3]. From a holding potential of −84 mV, cells were exposed to an initial saturating inhibitory stimulus, resulting in the closure of the MET channels, followed by a saturating excitatory step eliciting rapidly activating inward currents. Currents (averaged from 10 repetitions) before (black trace) and during (red trace) superfusion of 3 μM dTC **(A)**, 30 μM dTC **(B)**, 3 μM berbamine **(C)**, and 30 μM berbamine **(D)** were scaled and superimposed. Maximum transducer currents were; **(A)** −679 pA before and −369 pA during 3 μM dTC application, **(B)** −742 pA before and −137 pA during 30 μM dTC application, **(C)** −271 pA before and −211 pA during 3 μM berbamine application and **(D)** −271 pA before and −67 pA during 30 μM berbamine application. Both dTC and berbamine can be seen to act as open channel blockers from the decline in current size observed following the excitatory step. The currents during compound superfusion were fitted with single exponentials **(A)** τ = 6.9 ms **(B)** τ = 2.1 ms **(C)** τ = 3.2 ms **(D)** τ = 0.83 ms. **(E)** Entry rates into the OHC of extracellular dTC and berbamine as a function of membrane potential. At all membrane potentials berbamine enters the cell more rapidly than dTC. Rates calculated for 1 μM compound, 80 channels, p_open_ 0.1.

Large step stimuli were applied to the cells before and during superfusion of the compounds to confirm whether or not the channel is required to open before block can occur, i.e., whether the compounds act as open-channel blockers, as suggested by the MET currents recorded in response to sinusoidal stimuli (Figures 5A,B), and to quantify the time course of the block. Currents were recorded in response to a large force step before and during superfusion with different concentrations of each compound (3–30 μM; examples of recordings are shown in Figures 9A–D). In the absence of the blockers the force steps result in large, saturating MET currents showing minimal adaptation. Upon superfusion of either dTC or berbamine the currents activate with the same rapid time course, followed by an exponential decline in the currents, with the speed of the decline increasing with increasing concentration. This decline in the current following channel opening indicates that both compounds act as open-channel blockers and can only access their binding site once the channel is open. The time constants measured from the MET current decline allow for a calculation of the rate constants for entry into the channel from the extracellular side (*k*_*1*_; see Marcotti et al., 2005; van Netten and Kros, 2007). Mean values of the time constants were, for dTC, 6.7 ± 0.6 ms (*n* = 7 OHCs) for 3 μM and 2.1 ± 0.2 ms (*n* = 6) for 30 μM. For berbamine at 3 μM the time constant was 4.7 ± 0.4 ms (*n* = 10), at 10 μM 2.45 ± 0.15 (*n* = 5) and at 30 μM 0.97 ± 0.22 ms (*n* = 3). From these values, the entry rates into the hair cells can be calculated. At all potentials berbamine enters more avidly than dTC (Figure 9E).

### Effect of the compounds on the basolateral potassium currents in mouse outer hair cells

**Figure 10:**
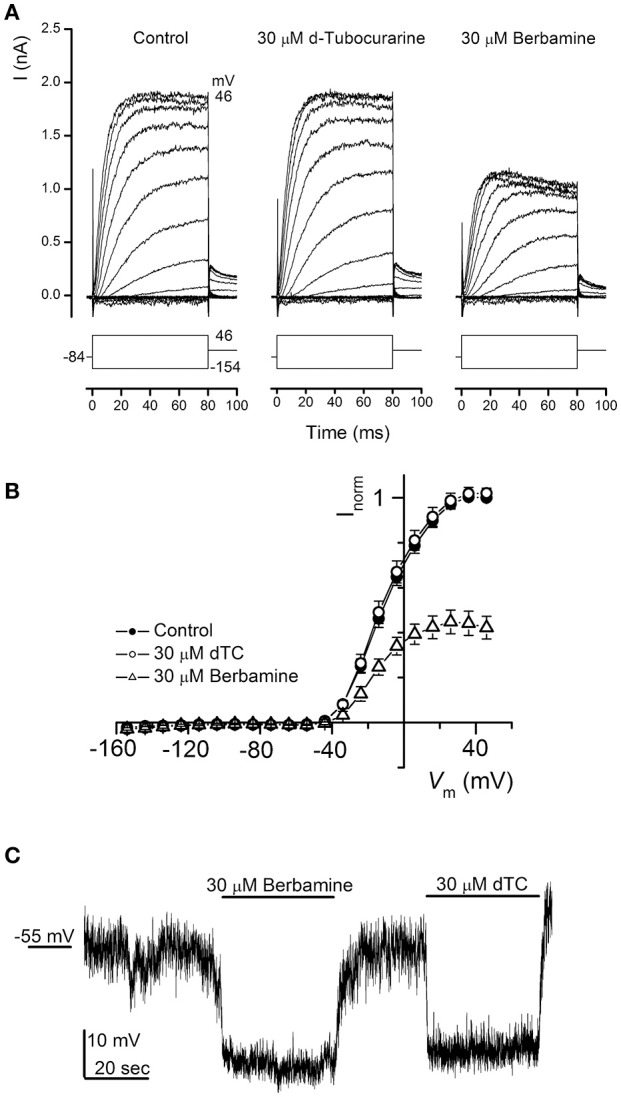
Basolateral potassium currents are blocked by berbamine but not by d-tubocurarine. **(A)** Currents from an OHC recorded before and during exposure to 30 μM dTC or berbamine reveal a decrease in current size during berbamine, but not dTC superfusion. Currents were recorded in response to 10 mV hyperpolarizing and depolarizing voltage steps from a holding potential of −84 mV. Schematic representations of the voltage step protocols are shown below each trace. The leakage currents have been subtracted but the membrane potentials have not been adjusted for the residual series resistance (3.0 MΩ). The capacitance of the cell was 6.7 pF. **(B)** Average normalized steady-state I-V curves for all cells before (closed symbols; *n* = 12) and during exposure to 30 μM dTC (open circles; *n* = 9) or 30 μM berbamine (open triangles; *n* = 8). **(C)** Monitoring membrane potential changes under current clamp revealed that exposure to both 30 μM berbamine and 30 μM dTC resulted in a hyperpolarization of the cell of more than 10 mV from the resting potential of −55 mV.

dTC is a known nicotinic antagonist that has also been shown to block various potassium channels including the apamin-sensitive potassium current in neurones (Goh and Pennefather, 1987), cloned small conductance calcium-activated potassium (SK) channels (Ishii et al., 1997) and a calcium-dependent potassium current in rat tumoral pituitary cells (Vacher et al., 1998). During the first postnatal week OHCs express a slow outward K^+^ current (I_K,neo_) activated at potentials positive to −50 mV (Marcotti and Kros, 1999). We therefore investigated whether dTC or berbamine could suppress this current and confer additional protection through causing a shift in resting membrane potential, with any depolarization of the cell potentially resulting in a reduced driving force for the positively charged aminoglycosides to enter the cells. Currents were elicited by applying a series of hyperpolarising and depolarizing voltage steps from the holding potential of −84 mV and currents recorded before and during exposure to 30 μM of each compound. Berbamine was found to substantially reduce I_K,neo_ whereas dTC had no effect at this concentration (Figure 10A). Average normalized current-voltage curves for all cells recorded from are shown (Figure 10B).

Notably, neither compound elicited a reduction in the resting membrane potential when it was perfused onto OHCs under current clamp at a concentration of 30 μM (Figure 10C). In fact the reverse was observed, with similar increases in resting membrane potential during both dTC and berbamine superfusion. This hyperpolarization, which would lead to an increase in the electrical force driving the aminoglycosides to enter the hair cells, eliminates changes in resting membrane potential as a potential protective mechanism for both dTC and berbamine.

### d-tubocurarine and berbamine reduce GTTR loading into cochlear hair cells

**Figure 11:**
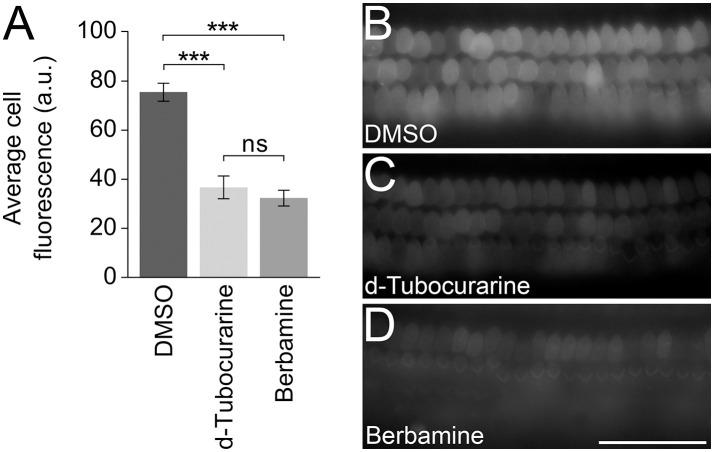
d-Tubocurarine and berbamine reduce the loading of GTTR. **(A)** dTC and berbamine significantly reduced the fluorescent intensity of 0.2 μM GTTR compared to DMSO controls (^***^p < 0.001). No significant difference was seen between the reduction by dTC and berbamine. Fluorescent intensity was measured in arbitrary units (a.u.). Error bars are ±SEM. **(B–D)** Representative images taken following 10 min GTTR exposure in DMSO, dTC and berbamine. Scale bar = 50 μm.

In order to test whether either compound could block or reduce the accumulation of gentamicin into the OHCs, mouse cochlear cultures were exposed to a low concentration (0.2 μM) of GTTR in the presence of a large molar excess (100 μM) of each compound for a short period of time to enable a quantification of the gentamicin uptake (Steyger et al., 2003). For a negative control, cultures were incubated in 0.2 μM GTTR and 1% DMSO alone. Following a 5 min pre-incubation with dTC, berbamine or DMSO alone and a subsequent 10 min co-incubation with 0.2 μM GTTR, a significant decrease in GTTR loading was seen in OHCs that were exposed to GTTR in the presence of either dTC or berbamine compared to the DMSO vehicle controls (Figures 11A–D). The reduction in GTTR labeling observed with the two compounds was similar and not significantly different.

## Discussion

The results of this study show that berbamine protects hair cells in mouse cochlear cultures from gentamicin toxicity, and that dTC can protect zebrafish lateral line hair cells from the toxic side effects of both neomycin and gentamicin. Furthermore, we confirm previous observations showing that dTC can protect mammalian hair cells from gentamicin (Alharazneh et al., 2011) and that berbamine can protect zebrafish hair cells from aminoglycoside toxicity (Kruger et al., 2016). The two alkaloids dTC and berbamine are therefore versatile otoprotectants that work in both fish and mammals.

Following on from this we set out to determine whether berbamine and dTC share the same mechanism of protection in mammals. The MET channels are the main entry site for the aminoglycosides into the hair cells (Marcotti et al., 2005) and a block of these channels has been suggested as the mechanism of protection for both berbamine in zebrafish lateral line hair cells (Kruger et al., 2016) and curare in rat OHCs (Alharazneh et al., 2011). Furthermore, previous studies have shown also that dTC is a MET channel blocker (Glowatzki et al., 1997; Farris et al., 2004). Our results clearly demonstrate that both compounds act as permeant blockers of the MET channel, rapidly and reversibly blocking the channels with similar half-blocking concentrations. Maximum block is seen at −118 mV for dTC and −94 mV for berbamine, with the block reducing at more hyperpolarized potentials, indicating both compounds can enter into the cells, albeit at a greatly reduced rate compared to the aminoglycoside DHS (Marcotti et al., 2005). For example, with the conditions chosen for Figure 9E (1 μM compound, 80 MET channels, p_open_ 0.1), the entry rates into the OHCs are 110 molecules/s for dTC and 205 molecules/s for berbamine, at a membrane potential of −150 mV. Taking the parameters for DHS permeation in the presence of 1.3 mM extracellular Ca^2+^ from Marcotti et al. (2005), the entry rate of 1 μM DHS into the cells would be some 1,130 molecules/s, an order of magnitude faster. For higher concentrations the entry rates started to saturate, so rates for dTC and berbamine can never approach those for DHS (e.g., for 100 μM rates were 319 molecules/s for dTC, 1107 molecules/s for berbamine and 11,460 molecules/s for DHS).

Whilst a previous study has reported that dTC is non-permeant and remains in the channel pore (Farris et al., 2004), this finding was based on studies in turtle auditory hair cells in which the cells were not hyperpolarized much beyond the potential at which we observe maximum block. The release of the block was therefore not observed. In our study cells were hyperpolarized to –164 mV, a potential at which a release of the block was clearly evident. This is a physiologically relevant membrane potential as the electrical driving force across the MET channels in the mammalian cochlea *in vivo* is generated by a positive endocochlear potential of +80 to +100 mV (Bosher and Warren, 1971) and a negative hair-cell resting potential of some −40 to −60 mV (Johnson et al., 2011). In turtle hair cells, Farris et al. reported *K*_*D*_ values for dTC block of 6.3 μM for the steady-state current and 16 μM for the peak current, values ~3–8 times higher than our (near steady-state) finding of 2.2 μM. They also calculated a Hill coefficient of 2 suggesting the cooperative binding of 2 dTC molecules in the channel pore as opposed to the negative cooperativity suggested by our finding of a Hill coefficient of 0.8. These observations imply marked differences between the turtle and mouse MET channels, highlighting the need for caution in interpreting results across species.

**Figure 12:**
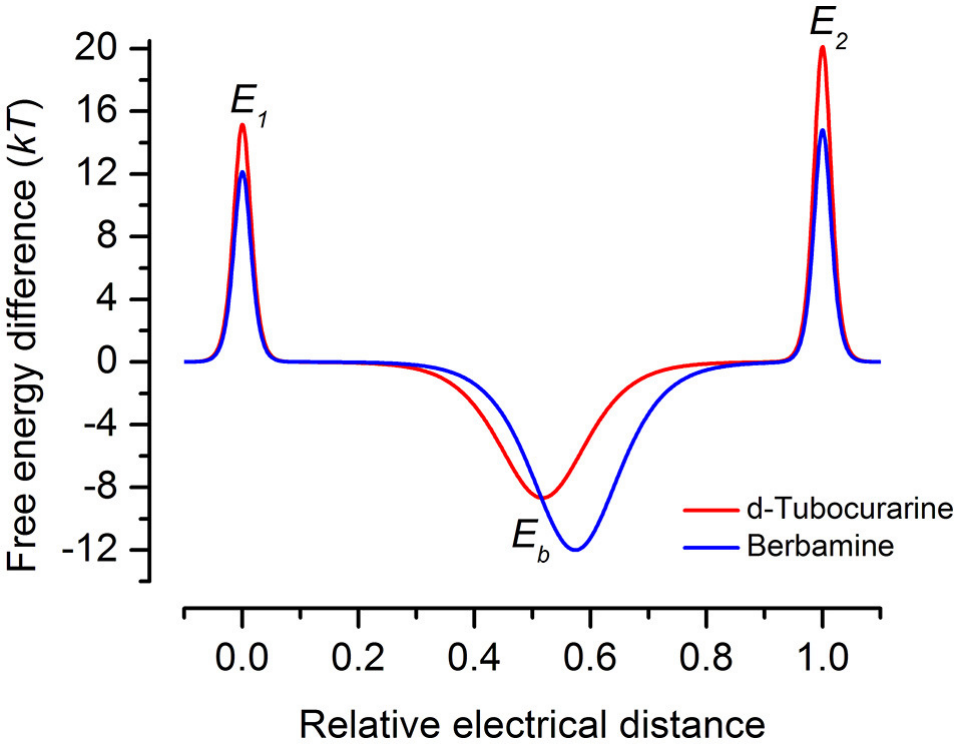
Energy profiles for MET channel permeation and block by extracellular d-tubocurarine and berbamine. Energy profiles calculated from fits to the fractional-block curves and kinetics of MET current block are shown. Values for the free energies of the binding site E_b_ and barriers E1 and E_2_ are shown in the absence of a voltage across the membrane (*V*_*m*_ = 0 mV). The voltage-independent extracellular barrier E_1_, at an electrical distance of zero, has a free energy of 15.16 kT for dTC and 12.14 kT for berbamine. E_b_ is −8.67 kT for dTC and −12.0 kT for berbamine. The binding sites, δ_*b*_, are located at an electrical distance from the extracellular side of 0.52 for dTC and 0.57 for berbamine. The intracellular barrier E_2_, positioned at an electrical distance of one, is 20.13 kT for dTC and 14.82 kT for berbamine.

Both dTC and berbamine significantly reduce the loading of GTTR into the OHCs. This observation, together with the knowledge that they are both MET channel blockers with a reasonably high affinity for the channel pore, strongly suggests that protection is conferred via a competitive block of the channels. Both compounds can, however, enter the cells so it is possible that there are alternative and/or additional intracellular targets. This seems unlikely though as dTC and berbamine provide protection against both neomycin and gentamicin in zebrafish, a species in which these aminoglycosides activate distinct cell death pathways (Owens et al., 2009; Coffin et al., 2013a,b) and may have different targets (Owens et al., 2008; Vlastis et al., 2012). Although berbamine and dTC are both permeant, open-channel blockers of the hair cell's MET channel that reduce GTTR loading into hair cells and protect against aminoglycoside toxicity, berbamine was found to be toxic to mammalian hair cells and other cell types in the developing organ of Corti at concentrations ≥30 μM. It also blocks the hair cell's basolateral K^+^ current I_K,neo_. As mentioned above, berbamine is more permeant than dTC, and the energy profiles (Figure 12) indicate substantial differences between their interactions with the MET channel, with dTC having higher entry and exit barriers. The latter feature would hinder its entry into the hair cells.

**Figure 13:**
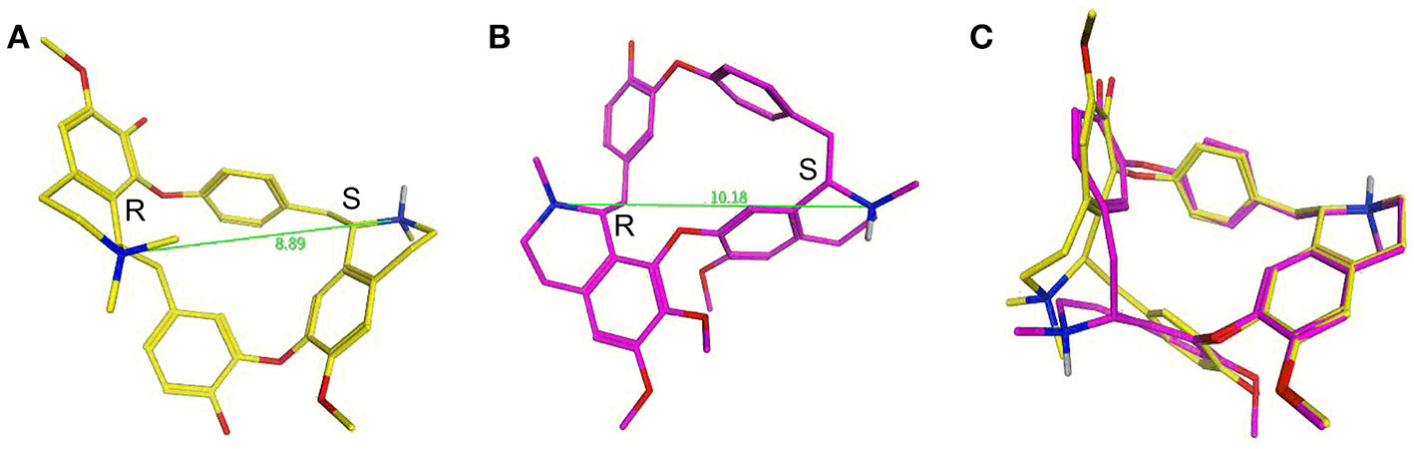
d-Tubocurarine and berbamine share similar chemical structures. **(A,B)** Energy-minimized structures showing the distance between the positive charges for dTC (8.89 Å; yellow) and berbamine (10.18 Å; magenta) respectively. **(C)** A flexible alignment with the structures not minimized reveals dTC and berbamine share striking structural similarities.

dTC is an alkaloid formed of two isoquinoline moieties linked via hydroxyl-benzyl groups to form an 18 atom macrocycle. It bears one fixed positive charge (quaternary nitrogen) and a pH dependent positive charge (tertiary nitrogen, calculated pKa ~8.0). Ionization simulation shows that at physiological pH ~80% of the molecule will bear two positive charges. The distance between the two positive charges of dTC (Figure 13A), with the stereochemistry of the carbon atoms next to the quaternary and the tertiary nitrogens being R and S respectively, is 8.89 Å. Whilst berbamine is an 18 atom macrocycle alkaloid that is structurally related to dTC and bears the same two isoquinoline moieties the latter are, however, linked differently. In comparison to dTC, berbamine does not bear any fixed positive charge but has instead two pH dependent ones, with the calculated pKa for the two tertiary nitrogens being ~7.4 and 8.2. Prediction of ionization status at physiological pH suggests that 50% of the molecules will bear two positive charges and ~40% only one positive charge. The distance between the two positive charges for berbamine, with the stereochemistry of the carbon atoms next to the two nitrogens being R and S (Figure 13B), is 10.18 Å, slightly higher than that in dTC. Flexible alignment of the two structures (Figure 13C) shows a good overall superimposition of the molecules; however, the isoquinoline moiety bearing the quaternary nitrogen of dTC and one of the two tertiary nitrogens of berbamine do not superimpose properly with the two nitrogens being 1.2 Å apart from each other.

Some of the differences between the structures of dTC and berbamine outlined above may explain why their properties and interactions with the MET channel are different. For example, the higher entry and exit energy barriers for dTC could tentatively be explained by the fact that a higher percentage of dTC molecules will bear two positive charges compared to berbamine molecules at physiological pH, thereby hindering the passage of dTC across the positive charges present at the mouth and exit of the channel (van Netten and Kros, 2007). Alternatively, the distance between the positive charges may critically determine the strength of interactions with the MET channel protein. As discussed earlier, dTC and berbamine are both less permeant than the aminoglycoside DHS (Marcotti et al., 2005), entering the cells at a substantially slower rate, by an order of magnitude. This may, in part, be explained by the fact that dTC and berbamine both have a more rigid structure, are less flexible compared to DHS, and may therefore be unable to adapt their conformation within the channel.

In the search for potential otoprotective compounds we have identified dTC as a promising lead compound for further investigation. dTC is a reasonably high-affinity MET channel blocker (*K*_*D*_ = 2.2 μM) that rapidly and reversibly blocks the channel. It has advantages over berbamine which include reduced permeability and a lack of toxicity up to 50 μM. By comparison berbamine had only a narrow range of protective concentrations in the cochlea, as it was found to be toxic at concentrations above 30 μM. Moreover, berbamine damaged OHCs when applied intracellularly at 1 mM. As berbamine was not toxic to neuromast hair cells, and protected these cells at lower concentrations than dTC, results from the zebrafish assay alone would have favored berbamine over dTC as a potential otoprotective compound. This points to the necessity to follow-up results from zebrafish screening with a mammalian otoprotection assay. One future approach would be to modify dTC in order to increase its affinity for the MET channel and eliminate its ability to enter the cells. In parallel one would need to reduce its action as a nicotinic antagonist to avoid blocking the middle ear reflex and the action of the olivocochlear bundle following trans-tympanic application.

## Author contributions

Participated in research design: NK, MD, EK, RH, SvN, SW, GR, CK. Conducted experiments: NK, MO, EK, GR. Performed data analysis: NK, MO, MD, EK, RH, CK. Wrote or contributed to the writing of the manuscript: NK, MO, MD, EK, SvN, GR, CK.

### Conflict of interest statement

The authors declare that the research was conducted in the absence of any commercial or financial relationships that could be construed as a potential conflict of interest.
